# Breast cancer incidence in Greek women in relation to ABO blood groups and Rh factor

**DOI:** 10.1186/1477-7800-6-14

**Published:** 2009-08-18

**Authors:** Michael Stamatakos, Konstantinos Kontzoglou, Panagiotis Safioleas, Constnatinos Safioleas, Christina Manti, Michael Safioleas

**Affiliations:** 1 2nd Department of Propaedeutic Surgery, University of Athens, School of Medicine, LAIKO General Hospital, Athens, Greece; 2 Department of Hematology, Thriassio General Hospital, Athens Greece

## Abstract

**Aim:**

To investigate the correlation between breast cancer in Greek women and ABO blood groups.

**Material-methods:**

In 166 female patients with breast cancer factors such as blood group, histological type, family history, presence or absence of nodal and/or distant metastases were examined. These patients had similar demographic, clinical, surgical, immunohistochemical, laboratory, and follow-up data and this group is representative of general population of women in Greece.

**Results:**

The ductal type of breast cancer was differentially distributed in blood groups Rh (+) (P ≤ 0.001). In patients with A (+) blood group the ductal type of breast cancer was present in 49.6% of cases, in relation to the other blood groups and in AB blood group the same type occurred rarely (3.6%). Rh (+) women with positive family history were more often found in A blood group. The relative risk of metastasis in Rh (-) patients was 4.2 times higher than that in Rh (+) patients. Among Rh (+) patients, the relative risk of metastasis was 1.29 times higher in A blood group than in other blood groups.

**Conclusion:**

Blood group A is often associated with ductal breast cancer (49.6%), in contrast to the other blood groups and particularly to blood group AB (3.6%). Blood group A and, particularly, A (-) has the worst prognosis of all.

## Introduction

The role of ABO/Rh blood group as a prognostic factor in breast cancer has been examined in the past; however, existing data are surprisingly few and the prognostic role of blood group remains controversial [1]. There is however evidence that ABO blood groups may have importance, since the antigens of blood groups seems to have a significant biological role in the immunological system, thereby promoting the development of some tumors, including breast cancer [2]. The aim of this study is to investigate the presence of a possible association between breast cancer and blood groups ABO and Rh.

## Materials and methods

Blood samples were collected from 166 women with breast cancer during their preoperative control and follow-up, following mastectomy, in Laiko General Hospital. These patients had similar demographic, clinical, surgical, immunohistochemical, laboratory, and follow up data. This group was representative of Greek women among the general population. Factors like family history, clinical/surgical findings, blood group, histological/immunohistochemical findings (including type of cancer, degree of malignancy, and hormone receptor status), and the presence of nodal and/or distant metastases were investigated. Three hundred women with benign breast diseases were used as a control group to allow statistical analysis. This control group is a representative group at national level in regard to the ABO blood group system and Rh status distribution.

Statistical analysis was performed by the Epidemiology Sector of the National Institution of Public Health. Descriptive statistics were calculated for all variables. Odds ratios with 95% of confidence intervals were generated in case that the essential statistical norms occurred and x^2 ^test, for possible association of examined factors and measurement was applied, and whenever possible, the relative risk (RR) was calculated, for each variable separately [3-5].

## Results

Mean age of patients was 55.31 years (range, 25 – 86 yrs). Tumor size ranged from 1 to 9 cm (mean, 2.77 cm). The most common type of breast cancer was ductal (n = 146), followed by combined (n = 11) and lobular (n = 9). Metastatic nodal disease was present in 85 patients, while 35 patients had distant metastases. Invasive breast cancer was diagnosed in 119 patients, while non-invasive in situ in 47 patients. Of all 166 patients of the study, 86 were progesterone and estrogen receptors positive (PR + and ER +), 15 patients were PR +, and 20 were ER +. Data regarding correlation between histological type of breast cancer, family history, presence of distal metastatic disease and ABO/Rh blood groups are presented in Tables 1, 2, 3. In table 4 the distribution of blood groups (ABO/Rh) in the control group is presented. No positive correlation between age, size of the lump, nodal metastases, degree of malignancy or presence of progesterone/estrogen receptors and ABO blood groups system and Rh status, was found. Ductal breast cancer was found more frequently in patients with A blood group (49.6%) in relation to the other blood groups (P ≤ 0.001), whereas it was much more rare in patients with AB blood group (3.6%) (Table 5). Rh (+) patients with a positive family history have more often A blood group and less often AB or B blood group (P ≤ 0.001) (Table 6). The relative risk in Rh (-) patients for distant metastasis was 4.2 times higher compared to Rh (+) patients (P ≤ 0.001) (Table 7). However, among Rh (+) patients, the relative risk for distant metastasis was 1.29 times higher in A blood group compared to other blood groups (Table 8). As far as distant metastases are concerned in relation to each Rh(+) ABO blood group, it seems that they occur more frequently in A blood group, comparing to the rest ABO blood groups (P ≤ 0.001) (Table 9).

**Table 1:** Blood groups ABO/Rh and Histological types of Breast Cancer.

	**Histological Types**		
	**Ductal**	**Lobular**	**Combined**
**Blood** **Groups**			
A Rh (+)	68	2	5
A Rh (-)	4	0	0
B Rh (+)	19	1	0
B Rh (-)	2	0	1
AB Rh (+)	5	2	0
AB Rh (-)	1	0	1
O Rh (+)	45	4	4
O Rh (-)	2	0	0

**Total**	**146**	**9**	**11**

**Table 2:** Blood groups ABO/Rh in women with family history and Breast Cancer.

	**Family History**	
	YES	NO
**Blood Groups**		
A Rh (+)	21	54
A Rh (-)	2	2
B Rh (+)	4	16
B Rh (-)	0	3
AB Rh (+)	3	5
AB Rh (-)	0	1
O Rh (+)	16	37
O Rh (-)	2	0

**Total**	**48**	**118**

**Table 3:** Blood groups ABO/Rh in women with Metastasis and Breast Cancer

	**Metastasis**	
**Blood Groups**	**YES**	**NO**
A Rh (+)	16	59
A Rh (-)	2	2
B Rh (+)	5	15
B Rh (-)	1	2
AB Rh (+)	2	6
AB Rh (-)	1	0
O Rh (+)	7	46
O Rh (-)	1	1

**Total**	**35**	**131**

**Table 4:** The distribution of 300 Persons/controls regarding Blood Groups ABO/Rh.

**Blood Groups**	**Persons/Controls**	**Percentage %**
A	130	43.3%
B	29	9.6%
AB	17	5.6%
O	124	41.3%

Rh (+)	267	85%
Rh (-)	33	15%

**Table 5:** Blood Groups ABO/Rh(+) and Histological Ductal type of Breast Cancer.

**Blood Groups**	**Histological Ductal Type**
A Rh (+)	68
B Rh (+)	19
AB Rh (+)	5
O Rh (+)	45

**Total**	**137**

**Table 6:** Blood Groups ABO/Rh(+) in women with Family History of Breast Cancer.

**Blood Groups**	**Family History**	
	YES	NO
A Rh(+)	21	54
Other Blood Groups Rh(+)	23	58

**Total**	**44**	**112**

**Table 7:** Rhezus Blood Groups in women with Metastasis of Breast Cancer.

**Rhezus Blood Groups**	**Metastasis**		**Total**
	YES	NO	
Rh (+)	30	126	**156**
Rh (-)	5	5	**10**

**Table 8:** Metastasis in A Rh (+) Blood Groups in relation to other Blood Groups.

**Blood Groups**	**Metastasis**		**Total**
	YES	NO	
A Rh (+)	16	59	**75**
Other Blood Groups Rh(+)	14	67	**81**

**Table 9:** Metastasis in Rh (+) in relation to ABO Blood Groups.

**Blood Groups ABO/Rh(+)**	**Metastasis**
A Rh(+)	16
B Rh(+)	5
AB Rh(+)	2
O Rh(+)	7

**Total**	**30**

An abnormal distribution of ductal histological type in Rh (+) patients was noted (P ≤ 0.001). Ductal breast cancer occurred more frequently in Rh (+) patients, regardless of the ABO blood group (P ≤ 0.001).

## Discussion

The role of genetic factors in the development of cancer is widely accepted. During the last two decades, the role of inheritance in breast tumorigenesis has been clearly established, mainly after the description of BRCA1/2 and other genes. Alexander in 1921 reported that patients with blood group B and AB were more vulnerable to develop malignancy which can be more aggressive than neoplasms occurring in patients with other blood groups [6]. Aird and Bentall found an association between blood group A and gastric cancer [7]. This was subsequently confirmed by other investigators, which showed a further association between blood group A and pernicious anemia [8-10]. Pandey et al showed an increased frequency of carcinoma of the gallbladder in blood groups A and AB [11]. Specifically regarding breast cancer, Vogel reported that ABO blood groups can potentially influence the prognosis, being an independent prognostic factor [9]. Other groups of investigators have also recognized ABO blood groups as a predisposing or prognostic factor in breast cancer [2,9,12-14]. Previous studies have shown that women with A blood group are generally prone to develop neoplasms with poor prognosis and aggressive biological behavior and that these women represent a significant percentage among breast cancer patients, higher than the actual percentage of A blood group among the general feminine population [10,12,13]. In contrast, women with O blood group may have some "protection" against the development of breast cancer; even when these women have breast cancer, prognosis is usually more favorable [12]. Women with AB blood group have similarities with the A blood group. In contrast, women with B blood group have similarities with women of O blood group, especially when no family history exists. An interesting observation of some investigators is that breast cancer patients with blood group B are at a higher risk of being re-affected by breast malignancy compared with women of other blood groups [15]. This may be partially due to the fact that women with blood group B have better prognosis.

This study showed that a positive family history is more commonly found in Rh (+) patients irrelevantly of blood groups ABO. Rh (+) women with positive family history are more often presented in blood group A and less often in blood groups AB and B. Ductal type occurs more frequently in Rh (+) patients regardless of the blood group ABO. In Rh (+) patients, ductal breast cancer is differentially distributed and is commonly observed in patients with blood group A. The relative risk of metastasis in Rh (-) patient was 4.2 times higher compared to Rh (+) patients. Among Rh (+) patients the relative risk of metastasis in blood group A was 1.29 times higher compared with the risk in the other blood groups. Of all blood groups, group A and especially Rh (-) group A was associated with the worse prognosis.

Based on these data, ABO/Rh blood group could be used as a prognostic factor in breast cancer patients. This use of blood groups is an interesting proposition, given that the determination of blood group requires a simple and cheap examination. However, further studies with larger number of patients are needed to clearly establish the role of ABO/Rh blood groups as a prognostic factor in breast cancer patients.

## Abbreviations

Rh: Rhezus; TNM: Tumor – Node – Metastasis; RR: Relative Risc; PR: Progesterone receptors; ER: Estrogen receptors; BRCA 1: gene for breast cancer 1; BRCA 2: gene for breast cancer 2.

## Competing interests

The authors declare that they have no competing interests.

## Authors' contributions

A copy of the written consent is available for review by the Editor-in-Chief of this journal' to the authors' contributions section. MS: Surgeon who performed the operations and edited part of the manuscript. KK: Surgeon who performed the operation and edited most of the manuscript. PS: Literature search and helped to prepare the draft. CS: Literature search and revision of bibliography. ChM: The physician who sun the blood tests. MS: Surgeon who performed the operations.

All authors have read and approved the final manuscript.
